# Supplementary figures and images for: No Change, No Life? What We Know about Phase Variation in Staphylococcus aureus
Source: Microorganisms. 2021 Jan 25;9(2):244. doi: 10.3390/microorganisms9020244 (PMC7911514; doi:10.3390/microorganisms9020244)

Fig. S1

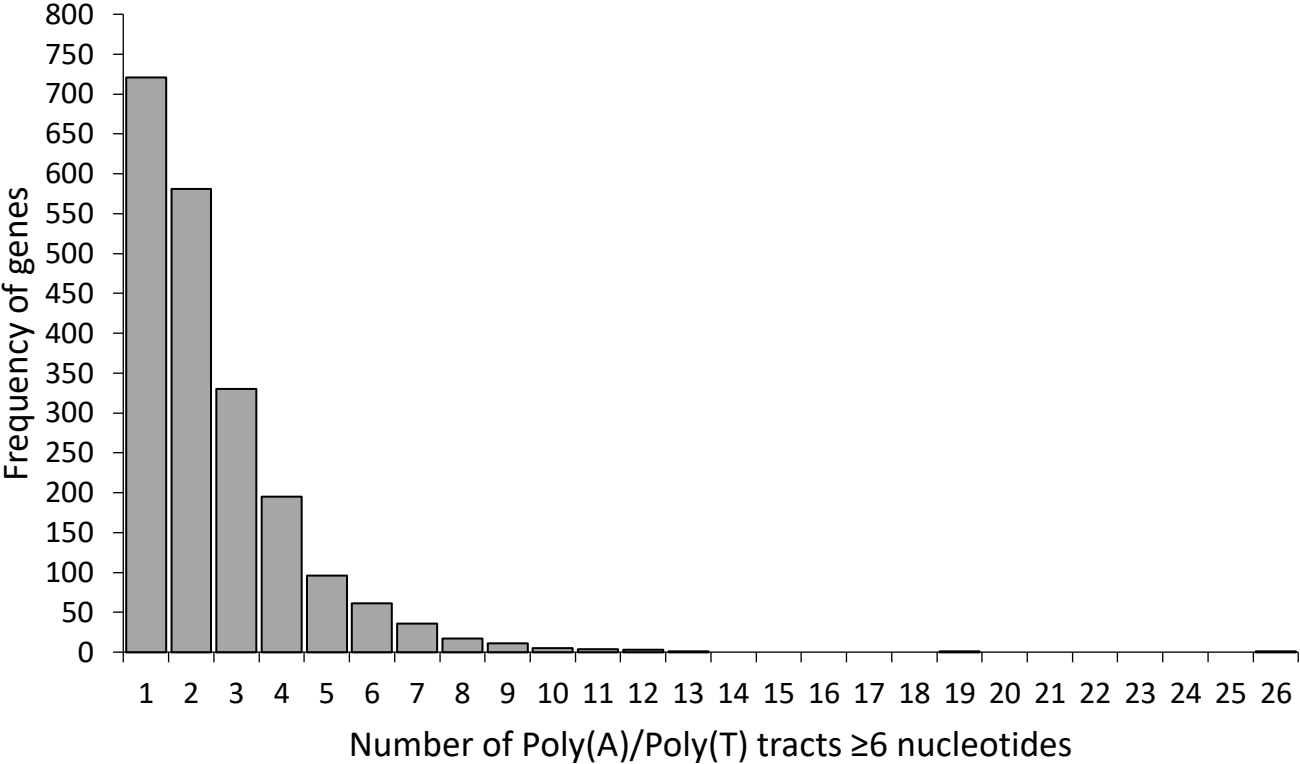

Supplement: Supplementary file 1 [file microorganisms-09-00244-s001.pdf]
